# The BigMove Intervention for People With Physical and Mental Health Conditions: A First Evaluation of Self-Perceived Health, Quality of Life, Coping and Mental and Social Functioning

**DOI:** 10.5334/ijic.8317

**Published:** 2024-08-05

**Authors:** Sabina van der Veen, Natalie Evans, Guy Widdershoven, Martijn Huisman

**Affiliations:** 1 Department of Ethics, Law and Humanities, Amsterdam UMC, Vrije Universiteit Amsterdam, Amsterdam Public Health Institute, Amsterdam, Netherlands; 2 Faculty of Social Sciences, Institute of Psychology, Health, Medical and Neuropsychology unit, Leiden University, Leiden, Netherlands; 3 Department of Epidemiology and Biostatistics, Amsterdam Public Health Research Institute, Amsterdam UMC, Amsterdam, The Netherlands; 4 Faculty of Social Sciences, Department of Sociology, VU University, Amsterdam, Netherlands

**Keywords:** integrated care, complex intervention, comorbidity, multimorbidity, self-perceived health, quality of life, coping, mental and social functioning

## Abstract

**Background:**

The BigMove intervention aims to improve the functioning and quality of life of people with physical and mental health conditions via an integrated care approach. This pilot study evaluates the impact of the intervention on self-perceived health (SPH), quality of life (QoL), active coping behaviour, and mental and social functioning.

**Methods:**

Data were analysed from N = 457 participants who had been referred to the intervention by their general practitioner (mean age 48.98 years; 76% female). Three patient-reported and one clinician-rated measures were used: SPH, QoL (MANSA), active coping behaviour (UPCC-ACT), mental and social functioning (HoNOS). Pre- and post-intervention measurements (from 2011 to 2018) were compared using paired-samples t-tests. Due to missing data, analyses were conducted with 205–257 participants per completed outcome. Associations with age and sex were assessed using repeated-measures ANOVA. Clinically relevant change was evaluated with the Edwards-Nunnally index and standard error of measurement (SEM) scores.

**Results:**

Post-intervention, there were statistically significant improvements for all outcomes (p < 0.0001) with moderate to large effect sizes (*d* = 0.41 to 1.02). The observed changes in outcomes can be considered as clinically relevant improvements.

**Conclusion:**

This pilot study provides preliminary evidence that the intervention has positive effects on SPH, QoL, active coping behaviour, and mental and social functioning.

## Introduction

Multimorbidity, the co-existence of more than one chronic health condition, is associated with a decrease in physical functioning, self-perceived health (SPH), quality of life (QoL) and an increase in the use of health services [1,2,3]. Additionally, people with chronic physical conditions are more prone to developing mental health conditions, such as depression, and conversely, people with mental health conditions are more at risk of developing long-term physical conditions [4,5,6]. Despite the interconnectedness of physical and mental health conditions, the interaction and the consequences of this interaction are usually overlooked in clinical practice [5]. Current treatment of physical and mental health is frequently organized to address single health conditions without paying attention to the interaction, resulting in fragmentation of care, poor communication between healthcare professionals, low satisfaction with care, and poor care outcomes [5,7].

To enhance care and address the overall care needs of people with multimorbidity while improving outcomes, a shift towards person-centred, integrated care approaches is needed [8,9]. Person-centred, integrated care was defined by the English National Voices Coalition of health and social care charities in 2013 as care in which ‘I can plan my care with people who work together to understand me and my carer(s), allow me to control, and bring together services to achieve the outcomes important to me.’ [10]. Moreover, to meet the needs of people with multimorbidity and improve healthcare outcomes, the World Health Organisation recommends that care should be organised within their community [11].

In 2003, the initial concept of the BigMove intervention was developed by a general practitioner and a physiotherapist providing care in a socio-economically disadvantaged and ethnically diverse neighbourhood in Amsterdam, who felt limited in their ability to influence persons with chronic health conditions to change their behaviour through conventional information and education. By 2011, the intervention was further refined, focusing on also addressing psychological functioning, given that many participants who participated in the initial interventions often dealt with mental health conditions next to physical problems. In response to the need to improve care for people with multimorbidity, the new version of the BigMove intervention was launched in 2011, offering person-centred, integrated, and community-based care for adults with a combination of physical and mental health conditions.

Central to the development of the intervention were two main premises. The first is that improving functioning, capabilities and Qol requires a combination of approaches and activities, and the second is that delivering integrated care requires strong collaboration among healthcare professionals. Aligned with person-centred care principles, a distinctive characteristic of the intervention was that participants were encouraged to assess their self-perceived functioning, set their own goals for improving their functioning, and specify action plans to achieve these goals. Adhering to the principle of integrated care, multiple healthcare professionals, collaborated in the execution of the intervention. Professionals were trained to enhance their coaching skills for both individual and group sessions, improve their understanding of the intervention’s structure, and clarify their roles within the interdisciplinary care team. This approach aimed to ensure that participants received holistic care, recognizing the interconnectedness of physical and mental health conditions. After the launch in 2011, the intervention was scaled up and offered at various places across the country. The intervention was conducted within the community or neighbourhood where participants lived, with local professionals providing support [9].

First participants were included in the intervention in December 2011. This study aims to conduct a first evaluation of the effects of the intervention. The first objective of this study is to investigate whether participants in the intervention demonstrate improvement in a range of outcomes, including SPH, QoL, active coping behaviour and mental and social functioning. The second objective is to examine whether changes in outcomes are clinically relevant.

## Methods

### Description of the intervention and participants

#### Intervention

The intervention aimed to improve the functioning, capabilities, and quality of life of people with a combination of physical and mental health conditions. The intervention was applied in more than 25 locations in the Netherlands. The planned duration for a participant was six months. Participants were referred to the intervention by their general practitioner if they had a combination of physical and mental health conditions and met the inclusion criteria. Each intervention group consisted of 12 to 15 people. Several healthcare professionals with diverse backgrounds and expertise collaborated in the execution of the intervention, such as mental health professionals, physical therapists, and general practitioners.

The design of the intervention combined individual and group sessions. Core elements of the intervention were motivational interviewing; functional goal setting (using the International Classification of Functioning, Disability and Health (ICF); cognitive behavioural therapy; group support; physical activity and enjoyment.

Participants attended two to five individual sessions with a BigMove professional, during which their progress was discussed, and previous individual goals were assessed and adjusted as necessary and three to ten individual CBT-sessions with a healthcare psychologist. The frequency was tailored to their specific needs for support. In the intervention, an e-health application was used, containing all ICF categories, which allowed for the assessment, registration and evaluation of the self-perceived functioning, as well as the formulation of related goals and action plans. The assessment was conducted on a tablet in the individual sessions with the BigMove professionals throughout the intervention. In follow-up sessions, again self-perceived functioning was assessed, and previous goals and action plans were evaluated to determine if they were still appropriate or required adjustment. Adjustment was necessary if goals had been achieved or had become less important to the participant. If a goal was no longer appropriate, it was no longer registered. We did not register whether goals were achieved. In addition to self-perceived functioning assessments, the application included a feature for administering the questionnaires for evaluation research. The application did not contain features for online sessions or messaging.

Next to individual sessions, weekly group sessions were organized. In the first group session, participants set group goals to foster cooperation and define joint activities. These goals were independent of the individual assessments based on the ICF. Halfway into the intervention the group goals were evaluated and adjusted if necessary. All group sessions included elements aimed at fostering social support within the group, physical activity, and enjoyment. However, the content of the group sessions was not predefined and followed the group preferences and process. The group could request guest sessions, for example, with a nutritionist or a yoga teacher.

During individual and group sessions, professionals aimed to facilitate reflection and interaction among participants with the goal of improving functioning, motivating participants to achieve their goals, and fostering changes in behaviour patterns. The group sessions were commonly organized in the gym of a physical therapy practice, a school sports hall, or a community centre. Groups also sometimes used local outdoor settings. The theoretical background, development, and design of the intervention are described in detail elsewhere [9].

### Participants

Inclusion criteria for referral to the intervention were:

- 18 years and older
- Diagnosed with a mental health condition (such as a mood, anxiety, behavioural or somatisation disorders) in combination with a chronic somatic condition.
- Experiencing a serious impact of these conditions on functioning in multiple areas of life.
- Physically capable of participating in the intervention.

Exclusion criteria were:

- Showing behaviour that disrupts group processes: threats, aggression, lack of impulse control, severe contact disturbance etc.
- Being in a psychotic state (e.g., delusions, hallucinations)
- Being at risk for criminal behaviour related to the mental health condition
- Being physically unable to participate in the intervention

From 2011 until 2019, 1976 people (1490 female, 486 male) participated in the intervention. Participants were most commonly referred to the intervention with a depressive disorder, an anxiety or panic disorder, a somatoform disorder and eating disorders in combination with diagnoses such as diabetes, COPD, obesity, or chronic fatigue syndrome.

We did not conduct an evaluation with separate arms for physical or mental health conditions because this was considered less appropriate since the intervention was designed to address the interconnectedness of physical and mental health and to intervene in an integrated manner.

### Measures

#### Self-perceived health (SPH)

SPH, or subjective health, refers to the judgment about one’s own health. SPH was measured by one question, which aims to synthesize important health factors for the individual [12]. The question is: ‘How is your health in general?’ The score is rated on a 5-point Likert scale ranging from 1–5 (very poor, poor, neither good/bad, good, very good). SPH is a strong predictor of functional decline and mortality [13]. SPH is relevant for people’s daily functioning as it has a direct influence on a person’s ability to participate in society and the labour force [14] and has a mediating effect on the relationship between multimorbidity and depression [15].

#### The Manchester Short Assessment of Quality of Life (MANSA)

QoL was measured using the Manchester Short Assessment of Quality of Life (MANSA). The MANSA is a brief instrument in which participants can rate their subjective satisfaction with life in general and with different aspects of life. In this study, the 12-item instrument was used. The score is rated on a 7-point Likert scale ranging from 1 (couldn’t be worse) to 7 (couldn’t be better). Higher scores indicate better QoL [16]. The Dutch translation of the scale has proven to be reliable and valid for several patient populations with mental health conditions [17]. While the EQL-D5 is commonly used for routine assessments of health-related quality of life, the MANSA was chosen because of its ability to provide a nuanced evaluation of subjective well-being and life satisfaction. MANSA’s initial validation focused on populations with mental health conditions, but it has been used in evaluations in a wider range of conditions, including populations with physical health conditions [18].

#### Utrecht Proactive Coping Competence, sub-scale ‘Active tackling’ (UPCC-ACT)

Active coping behaviour was measured with the Dutch Utrecht Proactive Coping Competences scale (UPCC). The UPCC consists of seven subscales, each corresponding to a different coping strategy. In this study, we only used the Active Tackling sub-scale, as the intervention specifically aims to improve participants’ sense of control and agency. This subscale consists of seven items and examines the participant’s ability to act actively when confronted with problems. The score is rated on a 4-point Likert scale from 1 (seldom/never) to 4 (very often). Higher scores indicate a more active coping approach [19]. Psychometric properties of the scale in several populations (older adults, patients with diabetes, patients with stroke and young adults) are good [20,21].

#### Health of the Nation Outcomes Scale (HoNOS)

Mental and social functioning was assessed with a clinician-rated scale, the HoNOS [22]. The scale assesses mental and social functioning in 12 items over four domains: (1) health and social behavioural problems, 2) organic problems, 3) psychological symptoms, and 4) social problems [23]. Outcomes are measured on a 5-point Likert scale from 0 (no problem within the period rated) to 4 (severe to very severe). It has acceptable reliability and validity and has proven to be suitable for tracking changes in functioning over time [24,25,26].

### Ethical Approval

Ethical assessment was done by the Institutional Review Board (IRB) of Amsterdam UMC, location VUmc (FWA00017598). The study was not considered to be subject to the Dutch Medical Research Involving Human Subject Act (Nr 2022-0690). The IRB checked informed consent and privacy issues. Participants of the intervention were informed and asked for consent to use the data for scientific purposes before the start of the intervention. The study received retrospective ethical approval. This retrospective approval was needed as there was no academic research partner involved in the project when the intervention was developed, and participants were asked for informed consent to use the data collected in the e-health app for research purposes.

### Design

The quasi-experimental study compared outcomes with a one-group pre-post design. Data were collected at two moments, at baseline and between 120 and 365 days in the intervention. The mental healthcare professional (a healthcare psychologist, clinical psychologist, or psychiatrist), who applied individual CBT in the intervention, was asked to complete the HoNOS. Participants were asked to complete SPH, MANSA and UPCC-ACT measures in the individual sessions with the BigMove professional who also guided the group sessions. The assessments were collected in the intervention’s e-health application [27].

### Statistical analyses

Raw data from the e-health application was transferred to CSV files and analysed with IBM Statistical Package for the Social Sciences (SPSS, Version 27). Participants with incorrect recorded age (<18) or of the location which was solely used for training purposes of professionals were excluded.

Two samples were included in the study. The first sample consisted of all participants (N = 612) with only a baseline measurement or with less than 120 days between two measurements. The intervention had a planned duration of six months. The second assessment could take place while the intervention was still ongoing, for instance because in a later individual session the e-health app was not used, or because the last individual session was scheduled before the last group session. A minimum of at least 120 days between intake and the second assessment was chosen, as all of the intervention’s essential elements had to be experienced over a substantial amount of time to reliably attribute to observed effects of the intervention. The second sample, the study sample, included all participants (N = 457) with at least two assessments, one before the intervention, and one between 120 and 365 days in the intervention. Inspection of the data on time in the intervention displayed skewness, with 83.2% of the included sample participating between 120–200 days. Given the skewness and concentration of the data, testing for interaction effects was considered unfeasible, as it would not provide a reliable basis for additional analysis.

The Cronbach’s alpha of the multi-item measurement instruments MANSA and UPCC-ACT indicated acceptable (>.0.7) internal consistency before and after a minimum of 120 days, respectively (0.81 and 0.86) and (0.83 and 0.83). The Cronbach’s alpha of the HoNOS had a score below 0.7 before (0.64) and above 0.7 (0.78) after a minimum of 120 days.

Scale scores for MANSA, UPCC-Act and HoNOS were computed and included when no more than one item had a missing value. The test re-test reliability was examined with the intraclass correlation coefficient (ICC), two-way mixed, absolute agreement for the patient-reported outcomes (SPH, MANSA and UPCC-ACT). For the HoNOS, the ICC was examined with two-way random, consistency. An ICC of <0.5, between 0.5 and 0.75, between 0.75 and 0.9, and greater than 0.90 indicates poor, moderate, good, and excellent reliability respectively [28,29]. The ICC for the SPH, MANSA, UPCC-ACT and HoNOS were respectively 0.55, 0.88, 0.89 and 0.80, indicating moderate (SPH) and good intraclass correlation agreement.

**Figure 1:**
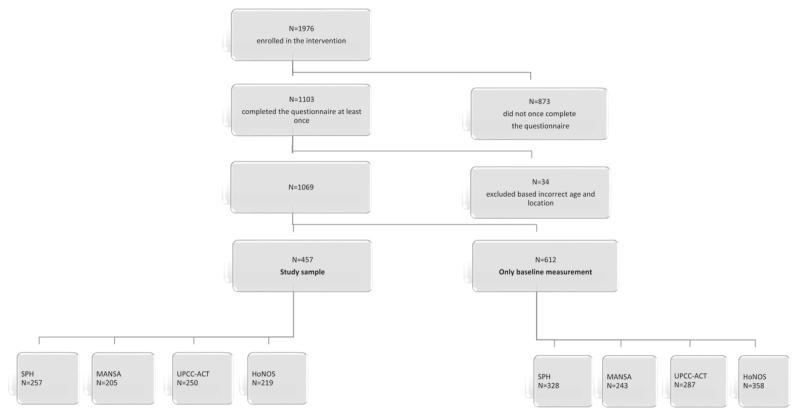
Flowchart.

The data were inspected for normality, outliers, and missing values. Regarding missing data, SPH had 28% missing data before and 41% missing data after. The scale scores of MANSA, UPCC-ACT and HoNOS displayed approximately the same percentages of missing data before and after, respectively 40%, 30% and 43% before and 41%, 45% and 44% after. The Little’s test, to test if the missing data is Missing Completely At Random (MCAR), was not significant, indicating that the missing data are at random. Therefore, we have decided to proceed with the analyses of complete data rather than use imputation methods, as these methods can lead to potential biases given the large number of missing data. The final number of participants included per outcome measure is given in Figure 1. Due to missing data, analyses were conducted with 205–257 participants per completed outcome. Several administrative issues could have caused missing data. As described above, the evaluations were administered in individual sessions with different professionals, who might not have been clearly instructed or not always followed instructions regarding the assessments of the questionnaires. Furthermore, measurements of the MANSA were included as of mid-2012. The HoNOS was added to the evaluation in 2013.

Participants were divided into three age groups, 1) below 45 years, 2) between 45 and 55 years, and 3) 55 years and older, to generate an equal distribution over the three groups and explore potential age differences in outcomes and changes in outcomes.

Descriptive statistics were used to describe potential differences between the baseline characteristics of patients who participated in the intervention but could not be included in the study because they had only one assessment, and of the participants of the study sample. Independent t-tests and chi-square analyses were performed for respectively continuous and categorical data to determine statistical differences between the baseline characteristics of both samples.

Changes between scores before and after a minimum of 120 days in the intervention were analysed using paired-samples t-tests; P < .05 was considered statistically significant. Cohen’s *d* was used as a standardised measure of effect (0.2 = small, 0.5 = moderate and 0.8 or more = large effect) [30]. A repeated measures ANOVA was conducted to explore potential differences for age groups and sex. Mauchly’s test was used to test the assumption of sphericity which indicates whether the variance of the differences between all possible pairs of related groups is equal. Partial eta squared (ηp2) was used to indicate the effect size, where 0.01 indicates a small effect, 0.06 a medium effect, and 0.14 a large effect. To assess clinical significance of changes within individuals two indexes were used. The first is the Edwards-Nunnally (EN) index [31], which is used to take regression to the mean into account. With the EN-index, an individual confidence interval is generated around the score at T0. If the score at T1 falls within this interval, it indicates that no significant clinical change in the outcome measure was found. If the score is either to the right or left of the interval, it can be interpreted as a clinical improvement or decline. The EN index is calculated with the following formula:


\[
{\mathrm{[rxx (Xpre - Mpre) + Mpre] \pm 2\ SDpre}}\surd {\mathrm{(1 - rxx)}}
\]

rxx = test-retest reliability of the measurement scale, Xpre = individual score at baseline (To), Mpre = mean of the sample score at baseline (To), SDpre = standard deviation of the sample scores at baseline (To).

As second index for clinically relevant improvement and decline, the standard error of measurement (SEM), was used [32]. The SEM was computed with the following formula: \[{\mathrm{SEM = SDpre}}\surd 1{\mathrm{- rxx}}\]. A change in outcome equal or greater than ± one SEM is considered clinically relevant improvement or decline as it represents the true change, beyond measurement error. One SEM to determine clinically relevant changes in this study is: SPH ± 0.53, MANSA ± 0.33, UPCC-ACT ± 0.21, and HoNOS ± 2.13.

## Results

The total number of participants enrolled in the intervention was 1976, of which 1490 (75.4%) were female. The mean age was 48.5 years. Of the 1976 participants, for 873 participants no data was collected. 1069 participants concluded the questionnaires at least once, that is: at baseline. 612 participants were included with only a baseline measurement or less than 120 days between two measurements. 457 participants had measurements before and after a minimum of 120 days in the intervention and were included in the sample for this study (Flowchart 1). Of the 457 participants included in the study sample, 349 (76%) were female. The mean age was 48.98 years ranging from 18 to 85 years.

Data from 612 non-sample participants with only a baseline assessment were investigated and were compared on baseline characteristics with the study sample (Table 1).

**Table 1:** Baseline characteristics of the study sample and the sample with only baseline information. M = Mean, SD = standard deviation. *^a^*chi-square test for categorical variables, independent for continuous variables.


BASELINE (T0) MEAN (SD, n)	STUDY SAMPLE AT BASELINE	NO VALID FOLLOW-UP MEASUREMENT	P-VALUE^a^

Number of participants (N = 966)	457	612	

Female/Male	349 (76%)/108	446 (73%)/166	0.196

Mean age (SD, range)	48.98 (12.6, 18–85)	48.92 (12.9, 20–89)	

Age groups			0.480

<45	160 (35%)	225 (36.8%)	

45–55	151 (33%)	181 (29.6%)	

>55	146 (32%)	206 (33.6%)	

**Outcome variables**			

SPH (M, SD, n)	2.84 (0,78, 328)	2.71 (0.81, 328)	0.044*

MANSA Scale score (M, SD, n)	4.18 (0.93, 275)	4.07 (1,08, 243)	0.200

UPCC-ACT Scale score (M, SD, n)	2.42 (0.61, 320)	2.39 (0.66, 287)	0.646

HoNOS Scale score (M, SD, n)	11.53 (4.71, 260)	10.69 (5.32, 358)	0.039*


Comparative analyses between the participants in the study sample and participants who only provided baseline information indicated that there were no statistically significant differences related to sex (χ2 (1), N = 1069, = 1.674, p = .196), age groups (χ2 (2), N = 1069 = 1.469, p = .480), the scale scores of the MANSA(t (516) = 1.283, p = .200) and UPCC-ACT (t (605) = .460, p = .646). However, the comparative analysis did reveal statistically significant differences between the samples for SPH (t (654) = 2.02, p = .044) and the scale score of the HoNOS (t (616) = 2.027, p = .039). The sample study scored significantly better on SPH but worse on the HoNOS than participants in the sample not included in the study, suggesting that participants in the study sample reported their overall health as slightly better on average than the non-study sample, but that their supervisors scored their mental and social functioning as worse, on average. Hence, there was not a strong indication that the study sample functioned uniformly better or worse than the group of excluded participants for whom we had data.

The average interval between the initial and subsequent assessment was 174 days, ranging from 120 to 363 days. Although the intervention was designed to have a six-month duration, it could take longer for all intakes to be completed and the intervention to begin, explaining the period longer than six months. Table 2 shows the pre-post intervention comparison with a paired-samples t-test of SPH, scale scores of the MANSA, UPCC-ACT, and HoNOS and the effect sizes of the observed change.

**Table 2:** Paired-samples T-Test. Outcomes at baseline and after a minimum of 120 days for SPH and scale scores of MANSA, UPCC-ACT and HoNOS. D = difference, SD = standard deviation, CI = confidence interval.


OUTCOME VARIABLES	BASELINE MEAN	>120 DAYS MEAN	D	SD	95% CI	P- VALUE	COHEN’S *d*

SPH (n = 257)	2.83	3.29	0.46	0.77	[–0.55, –0.37]	<.001	–0.60

MANSA (n = 205)	4.16	4.79	0.63	0.72	[–0.73, –0.53]	<.001	–0.87

UPCC-ACT (n = 250)	2.42	2.63	0.21	0.51	[–0.27, –0.15]	<.001	–0.41

HoNOS (n = 219)	11.65	7.09	–4.56	4.48	[3.96, 5.16]	<.001	1.02


The changes in outcome scores of the SPH and scale scores of the MANSA, UPCC-ACT and HoNOS after a minimum of 120 days in the intervention were all statistically significant with moderate (0.41, 0.60) to large effect sizes (0.87, 1.02), suggesting an improvement in the outcomes during the course of the intervention. Before the intervention, based on examination of the item scores of the MANSA, participants were least satisfied with their life as a whole, day-to-day activities, physical health and mental health, social life, sex life and financial situation and most satisfied with their accommodation, living situation, personal safety, family, and personal relations (See Appendix 1). The satisfaction with mental health increased most with a mean change of 1.26 after a minimum of 120 days in the intervention.

Most items of the UPCC-ACT had comparable mean scores at baseline, except for the item ‘I see problems as a challenge’, which scored lower than the other items before but also after a minimum of 120 days. The items ‘I stay calm in difficult situations’ and ‘I work purposefully to solve a problem’ demonstrated the largest mean change score at T1.

At baseline, the items ‘Physical Impairment’, ‘Depressed Mood’, ‘Other Mental or Behavioural Problems’, and ‘Problems with Relationships’ of the HoNOS had the highest mean scores, demonstrating relatively poor functioning on these items. At T1, the mean score for these items decreased more than those of other items, indicating relatively large improvement in these domains of functioning. The mean scores for the items ‘Self-harm’, ‘Hallucinations/delusions’ and ‘Problems with living conditions’ did not change significantly between T0 and T1.

To compare the change in outcomes for sex and age groups, repeated-measures ANOVA was used. Mauchly’s test indicated that the assumption of sphericity had not been violated. In Table 3, results are shown for the repeated measures ANOVA for sex. The results show no significant association between sex and outcomes for the SPH, MANSA and UPCC-ACT. The partial eta squared for UPCC-ACT did however indicate a small effect.

**Table 3:** Repeated measures ANOVA of outcomes by sex.


OUTCOME VARIABLES	SEX	BASELINE MEAN (SD)	>120 DAYS MEAN (SD)	F	P- VALUE	ηP2

SPH (n = 257)	Female (n = 186)	2.90 (0.74)	3.33 (0.63)	81.8	0.326	0.004

	Male (n = 71)	2.65 (0.88)	3.18 (0.78)			

MANSA (n = 205)	Female (n = 145)	4.26 (0.91)	4.88 (0.86)	129.8	0.895	0.000

	Male (n = 60)	3.92 (0.98)	4.55 (1.02)			

UPCC-ACT (n = 250)	Female (n = 182)	2.42 (0.63)	2.59 (0.55)	42.4	0.118	0.010

	Male (n = 68)	2.44 (0.67)	2.73 (0.58)			

HoNOS (n = 219)	Female (n = 174)	11.13 (4.21)	6.89 (4.19)	182.4	0.037	0.020

	Male (n = 45)	13.67 (6.13)	7.87 (5.84)			


Male participants had lower mean scores at T0 and T1 on the outcomes of SPH and MANSA than female participants, indicating they perceived their health and Qol as worse. The association between outcomes on the HoNOS indicated that male participants had statistically significant higher scores, indicating worse mental and social functioning, with a small effect, suggesting clinicians assessed male participants to be more severely impacted by their conditions.

In Table 4, results are shown for the repeated measures ANOVA by age groups. The results indicate that there was no significant association between the age groups and outcomes, however partial eta squared of the MANSA indicated a small effect. Mean outcomes of the age group 45–55 indicated poorer SPH, QoL and mental and social functioning both at baseline and after the intervention.

**Table 4:** Repeated measures ANOVA of outcomes by age group <45, 45–55, >55.


OUTCOME VARIABLES	AGE GROUPS	BASELINE MEAN (SD)	>120 DAYS MEAN (SD)	F	P- VALUE	ηP2

SPH (n = 257)	Age <45 (n = 93)	2.80 (0.82)	3.32 (0.71)	90.6	0.361	0.008

	Age 45–55 (n = 84)	2.74 (0.78)	3.21 (0.73)			

	Age >55 (n = 80)	2.96 (0.75)	3.32 (0.57)			

MANSA (n = 205)	Age <45 (n = 74)	4.20 (0.86)	4.74 (0.95)	157.1	0.370	0.010

	Age 45–55 (n = 65)	3.93 (1.01)	4.56 (1.02)			

	Age >55 (n = 66)	4.33 (0.91)	5.05 (0.70)			

UPCC-ACT (n = 250)	Age <45 (n = 90)	2.37 (0.63)	2.62 (0.59)	41.0	0.309	0.009

	Age 45–55 (n = 82)	2.43 (0.66)	2.66 (0.53)			

	Age >55 (n = 78)	2.48 (0.63)	2.62 (0.55)			

HoNOS (n = 219)	Age <45 (n = 76)	11.51 (4.69)	7.11 (4.42)	222.9	0.928	0.001

	Age 45–55 (n = 81)	12.35 (5.36)	7.74 (5.16)			

	Age >55 (n = 62)	10.92 (3.90)	6.23 (3.78)			


The results for clinically relevant changes based on the EN-index and the SEM scores are shown in Table 5.

**Table 5:** Individual clinically relevant change based on EN-index and the SEM. CI = Confidence interval.


CLINICALLY RELEVANT CHANGE (N, CI EN-INDEX)	EN- INDEX IMPROVEMENT	EN-INDEX STABLE	EN-INDEX DECLINE	SEM IMPROVEMENT	SEM STABLE	SEM DECLINE

SPH (n = 257) (1.78–3.89)	73 (28.4%)	184 (71.6%)	0 (0%)	117 (45.5%)	122 (47.5%)	18 (7%)

MANSA (n = 205) (3.53–4.84)	92 (44.9%)	110 (53.7%)	3 (1.5%)	138 (67.3%)	47 (22.9%)	20 (9.8%)

UPCC-ACT (n = 250) (2.00–2.84)	76 (30.4%)	154 61.6%)	20 (7.8%)	120 (48%)	81 (32.4%)	49 (19.6%)

HoNOS (n = 219) (15.59–7.58)	124 (56.6%)	90 (41.1%)	5 (2.3%)	146 (66.7%)	62 (28.3%)	11 (5%)


The two methods for determining clinically relevant changes, the EN-index, and the SEM, resulted in different percentages for clinical change. Based on the EN index, clinically relevant improvement on the instruments ranged between 28.4% and 56.6%, whereas between 0% and 7.8% clinically declined. The SEM indicated that between 45.5% and 67.3% of the participants improved and between 5% and 19.6% declined. Changes in QoL and mental and social functioning scores indicated higher percentages of clinically relevant improvement (MANSA 44.9%–67.3% and HoNOS 55.6%–66.7%) than the changes in SPH and active coping behaviour outcomes (SPH 28.4%–45.5% and UPCC-ACT 30.4%–48%).

## Discussion

This study presents a first repeated-measures pilot evaluation of the BigMove intervention, a person-centred, integrated and community-based care approach for people with physical and mental health conditions. The results of this study indicate that participants of the intervention showed significant improvement in SPH, QoL, active coping behaviour and mental and social functioning. To a considerable extent, the observed changes in outcomes can be considered as indicators of clinically relevant improvement. The essential elements of the intervention were intended to jointly facilitate behaviour change. The specific contribution of each individual elements to the outcomes was not investigated as our study focused on assessing their collective impact as they were designed to be offered in synergy.

### Interpretation of the findings

The significant improvements found in this study may be an indication that the intervention is a promising approach to address these people’s needs and improve outcomes for persons with chronic physical and mental health conditions. The moderate to large effect sizes observed across all outcomes further support the innovative approach, although it is important to consider that the HoNOS, being a clinician-rated instrument, carries the risk of overstating intervention outcomes [33].

In comparison to the other measures, the outcome of the UPCC-ACT is the least positive. The moderate effect size for active coping behaviour and the largely clinically stable outcomes aligns with earlier findings that improvements in coping styles often seem difficult to achieve [34]. Furthermore, the mechanisms of interventions to improve coping are still unclear [35]. It might be helpful to focus on mechanisms to improve proactive coping behaviour in the future development of the intervention as effective coping behaviour might support individuals to maintain behaviour change when confronted with behavioural barriers [36].

The majority of participants in both the study sample, those with only a valid baseline measurement as well as all participants in the intervention, were female. Despite females being more at risk of developing multimorbidity [37], our findings indicated that male participants in the intervention experienced more severe impacts on the outcomes from their health conditions. The intervention was open to all adults above 18, resulting in a broad age range among participants. Despite this variability, no significant associations were found between outcomes and age groups. These findings suggest that the intervention is broadly applicable across different age groups. However, further research is needed to understand the underlying factors for the observed differences in outcomes for male and female participants.

Baseline assessments indicated that participants’ SPH, QOL and mental and social functioning were significantly impacted when they enrolled in the intervention. Participants rated their SPH between poor and fair and the scale score of the MANSA was 4.16, which is lower than the reference score of 4.29 for patients with psychiatric conditions in the Netherlands [17]. The scale score of the HoNOS at baseline was 11.65 which qualifies as high for Dutch outpatients receiving ambulatory care [38]. These baseline scores suggest that participants were experiencing substantial impacts of their health conditions on various life domains. These baseline findings align with a study on the association between multimorbidity and SPH in older adults in Europe, which found that multimorbidity combinations that included high depressive symptoms were associated with an increased risk of perceiving poor SPH and increased rates of disability in daily life [3]. It is important to note that the findings of this study may not be generalisable to populations less affected by their health conditions. This factor should be considered when further developing the intervention, potentially necessitating adaptations tailored to the impact of health conditions at baseline, for example, in duration, intensity and design.

The large effect sizes for QoL and mental and social functioning found in this study are in contrast with findings of a systematic review of RCTs on interventions for people with multimorbidity in primary and community-based settings, which found small to no effects of the included interventions on QoL and functioning [39]. However, our study had an observational design, and consequently does not allow for direct comparison with RCTs. We did not find any observational and quasi-experimental studies with comparable objectives, outcomes, and designs to directly compare our findings.

While this study presented promising results, it should be noted that it is an exploratory pilot evaluation with no control group. As a result, caution is needed regarding the conclusion, given the scope for selection bias and lack of a control group.

### Strengths and limitations

A strength of the evaluation is that it included several patient-reported outcomes in combination with a clinician-rated outcome. Furthermore, the study included two methods to assess clinically relevant changes.

There are several limitations to be considered. First, the findings of this study apply to participants who responded to the questionnaire at least twice, that is before and after a minimum of 120 days of the intervention. Of the 1976 participants, only 457 participants were included in the study sample and of these, many had substantial information missing from one or more of the outcomes. Due to missing data, analyses were conducted between 205–257 participants per completed outcome. A possible explanation might be that not all the professionals involved in the intervention offered the questionnaires to participants. This results in a ‘convenience sample’, which poses a considerable risk of bias. Furthermore, while there are no large differences between the study sample and the sample of those of whom only measurements at T0 are available, it is unclear whether the study sample is representative of the entire group of participants in the intervention. Furthermore, this study lacks data on intervention attendance, which we acknowledge could potentially influence the outcomes.

Another limitation of the study is that it capitalised on the opportunity to examine data from an intervention that was utilized in clinical practice, where the main priority was to generate immediate benefit for participants, rather than on adhering to the gold standard for intervention studies. This was not a randomised study, nor a controlled one. Besides, we could only include a small subsample of all participants in the analyses and were only able to examine the influence of a small number of covariates such as sex, age and baseline scores. Other factors, such as socioeconomic status or disease duration that could influence the outcomes were not available. Also, no a priori power calculation was performed. Future research on the intervention should consider including a control group, such as those referred to the programme and on a waiting list or a care-as-usual approach. Furthermore, it could be improved by following the guidelines for evaluating complex interventions to assess the effectiveness of the intervention [40] and employing novel methods which are suitable to capture changes in functioning from the perspective of the person. And lastly, we did not register whether goals were achieved. While goal attainment can be a valuable outcome measure, the e-health app focused on assessing functioning and formulating new goals, rather than checking whether prior goals were reached. The idea behind this is that goal setting is more important than determining whether goals have been met, since functioning can also improve when specific goals are not met. Further details on changes in goal-setting are addressed in a forthcoming study.

Despite its limitations, the pilot evaluation offers exploratory insights in a research area currently lacking evidence. The analysis of clinically relevant changes, based on SEM and Edward Nunnally, demonstrates the potential effectiveness of the intervention, providing a basis for future research.

## Conclusion

The BigMove intervention offers innovative, person-centred, integrated, and community-based care for adults with a combination of physical and mental health conditions. This study is a first evaluation of the outcomes on SPH, QoL, active coping behaviour, and mental and social functioning of participants before and after at least 120 days in the intervention. Before the intervention, participants reported poor to fair self-perceived health and low quality of life and active coping behaviour, and clinicians indicated mental and social functioning was markedly impacted. After at least 120 days in the intervention, significant improvements in all outcomes were observed, regardless of sex or age, with moderate to large effect sizes. Also, most of these improvements seem to be clinically relevant. Further research is needed to determine how the interplay of the essential elements in the intervention and the efficacy of individual elements contribute to positive outcomes.

## Additional File

The additional file for this article can be found as follows:

10.5334/ijic.8317.s1 Appendix 1. Paired-samples T-Test Outcomes of scale and items scores at baseline and after minimum of 120 days for SPH and scale scores of MANSA, UPCC-ACT and HoNOS.
