# Supplementary material for: The BigMove Intervention for People With Physical and Mental Health Conditions: A First Evaluation of Self-Perceived Health, Quality of Life, Coping and Mental and Social Functioning
Source: Int J Integr Care. 2024 Aug 5;24(1):12. doi: 10.5334/ijic.8317 (PMC11312720; doi:10.5334/ijic.8317)
Supplement: Appendix 1. — Paired-samples T-Test Outcomes of scale and items scores at baseline and after minimum of 120 days for SPH and scale scores of MANSA, UPCC-ACT and HoNOS. [file ijic-24-3-8317-s1.pdf]

Appendix 1.

Paired-samples T-Test Outcomes of scale and items scores at baseline and after minimum of 120 days for SPH and scale scores of MANSA, UPCC-ACT and HoNOS

**Outcome variables**	**M Baseline**	**M > 120 days**	**D**	**SD**	**95% CI**		**P- Value**
**SPH (n= 257)**	2.83	3.29	0.46	0.77	-0.55	-0.37	.000
**MANSA-12 scale score (n=205)**	4.16	4.79	0.63	0.72	-0.73	-0.53	.000
1. Life as a whole (n=220)	3.49	4.51	1.02	1.35	-1.21	-0.85	.000
2. Accommodation (n=224)	4.90	5.33	0.43	1.40	-0.62	-0.25	.000
3. Living situation (n=222)	5.02	5.43	0.41	1.30	-0.58	-0.23	.000
4. Day to day activities (n=220)	3.58	4.53	0.95	1.54	-1.16	-0.75	.000
5. Physical health (n=222)	2.83	3.87	1.04	1.50	-1.24	-0.84	.000
6. Mental health (n=228)	3.05	4.31	1.26	1.46	-1.45	-1.07	.000
7. Personal safety (n=223)	4.61	5.24	0.63	1.45	-0.82	-0.44	.000
8. Social life (n=219)	3.97	4.79	0.82	1.49	-1.02	-0.63	.000
9. Family (n=223)	4.55	5.04	0.49	1.39	-0.67	-0.31	.000
10. Personal relationship (n=223)	4.70	5.17	0.47	1.22	-0.62	-0.30	.000
11. Sex life (n=220)	3.92	4.50	0.58	1.55	-0.78	-0.37	.000
12. Financial situation (n=226)	3.73	4.37	0.64	1.51	-0.84	-0.45	.000

M=Mean. D= difference, SD= standard deviation, CI= confidence interval.

**Outcome variables**	**M Baseline**	**M > 120 days**	**D**	**SD**	**95% CI**		**P-Value**
**UPCC—ACT Scale score (n=250)**	2.42	2.63	0.21	0.51	-0.27	-0.15	.000
1. I intervene immediately if there are difficulties (n=251)	2.46	2.66	0.20	0.93	-0.31	-0.08	.001
2. I see problems as a challenge (n=255)	1.95	2.24	0.29	0.85	-0.40	-0.19	.000
3. I'm looking at a problem from all sides (n=253)	2.68	2.85	0.17	0.88	-0.28	-0.07	.002
4. I stay calm in difficult situations (n=250)	2.26	2.48	0.22	0.80	-0.32	-0.12	.000
5. I think of different ways to solve a problem (n=252)	2.62	2.80	0.18	0.87	-0.29	-0.07	.001
6. I work purposefully to solve a problem (n=251)	2.43	2.67	0.24	0.85	-0.35	0.14	.000
7. I'll put things in order first (n=252)	2.57	2.76	0.19	0.80	-0.29	-0.09	.000

M=Mean. D= difference, SD= standard deviation, CI= confidence interval.

Outcome variables	M Baseline	M > 120 days	D	SD	95% CI		P-Value
**HoNOS Scale score (n=219)**	11.65	7.09	-4.56	4.48	3.96	5.16	.000
1. Aggression/ overactivity/ n=221	0.96	0.45	-0.51	0.92	0.39	0.64	.000
2. Self-harm n=219	0.17	0.12	-0.05	0.41	-0.004	0.11	.070*
3. Substance abuse n=220	0.24	0.14	-0.10	0.45	0.04	0.16	.001
4. Cognitive impairment n=220	0.96	0.59	-0.37	0.86	0.26	0.49	.000
5. Physical impairment n=219	2.01	1.43	-0.58	0.92	0.45	0.70	.000
6. Hallucinations/delusions n=215	0.15	0.09	-0.06	0.46	-0.003	0.12	0.063*
7. Depressed mood n=219	1.94	1.11	-0.83	0.99	0.69	0.96	.000
8. Other mental/behaviour problems n=219	1.99	1.21	-0.78	0.98	0.66	0.92	.000
9. Problems with relationships n=219	1.63	0.97	-0.66	0.95	0.54	0.79	.000
10. Problems in daily living n=214	0.58	0.35	-0.23	0.73	0.14	0.34	.000
11. Problems with living conditions n=218	0.27	0.23	-0.04	0.56	-.04	0.11	.333*
12. Problems with occupation/ activities n=219	0.72	0.41	-0.31	0.81	0.20	0.42	.000

M=Mean. D= difference, SD= standard deviation, CI= confidence interval.
